# Public health services knowledge and utilization among immigrants in Greece: a cross-sectional study

**DOI:** 10.1186/1472-6963-13-350

**Published:** 2013-09-13

**Authors:** Petros Galanis, Panayiota Sourtzi, Thalia Bellali, Mamas Theodorou, Ioanna Karamitri, Olga Siskou, Giorgos Charalambous, Daphne Kaitelidou

**Affiliations:** 1 Center for Health Services Management and Evaluation, Faculty of Nursing, University of Athens, Athens, Greece; 2 Department of Public Health, Associate Professor, Faculty of Nursing, University of Athens, Athens, Greece; 3 Department of Nursing, Alexandreio Technological Educational Institute, Thessaloniki, Greece; 4 Faculty of Economic Sciences and Management, Open University of Cyprus, Nicosia, Cyprus; 5 General Hospital of Kalamata, Messinia, Greece, Hellenic Open University, Patras, Greece; 6 Center for Health Services Management and Evaluation, Faculty of Nursing, University of Athens, Athens, Greece; 7 Emergency Department, Hippocratio Hospital of Athens, Athens, Greece

**Keywords:** Access, Greece, Immigrants, Knowledge, Public health services

## Abstract

**Background:**

During the 90s, Greece has been transformed to a host country for immigrants mostly from the Balkans and Eastern European Countries, who currently constitute approximately 9% of the total population. Despite the increasing number of the immigrants, little is known about their health status and their accessibility to healthcare services. This study aimed to explore the perceived barriers to access and utilization of healthcare services by immigrants in Greece.

**Methods:**

A pilot cross-sectional study was conducted from January to April 2012 in Athens, Greece. The study population consisted of 191 immigrants who were living in Greece for less than 10 years. We developed a questionnaire that included information about sociodemographic characteristics, health status, public health services knowledge and utilization and difficulties in health services access. Statistical analysis included Pearson’s ×^2^ test, ×^2^ test for trend, Student’s t-test, analysis of variance and Pearson’s correlation coefficient.

**Results:**

Only 20.4% of the participants reported that they had a good/very good degree of knowledge about public health services in Greece. A considerable percentage (62.3%) of the participants needed at least once to use health services but they could not afford it, during the last year, while 49.7% used public health services in the last 12 months in Greece. Among the most important problems were long waiting times in hospitals, difficulties in communication with health professionals and high cost of health care. Increased ability to speak Greek was associated with increased health services knowledge (p<0.001). Increased family monthly income was also associated with less difficulties in accessing health services (p<0.001).

**Conclusions:**

The empowerment and facilitation of health care access for immigrants in Greece is necessary. Depending on the needs of the migrant population, simple measures such as comprehensive information regarding the available health services and the terms for accessibility is an important step towards enabling better access to needed services.

## Background

According to the WHO European Region Health 2020 policy framework [1], successful governments may achieve real improvements in health if they work across government to improve health for all and reduce health inequalities. Many groups, with immigrants being one of them, have been left behind and, in many instances, as economies falter, health inequalities are growing within and between countries. Access for all, to high-quality and affordable care is considered one of the milestones towards an equitable, sustainable and of high quality health system.

Greece has been traditionally a country which sent immigrants to rapidly developing countries. However, since the early ‘90s it has transformed into a country which hosts immigrants, mainly from the Balkans [2]. In 2009, 958.000 immigrants (both documented and undocumented) were estimated to be living in Greece comprising 9% of the population [3]. Most of the immigrants are coming from Albania (58%) and countries from Eastern Europe (Ukraine, Moldavia, Georgia etc.) at a percentage of (14%) while immigrants from Asia and Africa are estimated to be 10% of migrant population.

Evidence shows that immigrants are more vulnerable to social and economic disadvantage, something that affects health outcomes as well as access to health care [4,5]. In particular, social exclusion is closely linked to migration [6]. Socially excluded are defined by the same authors, as those who are marginalised and stigmatised due to their distinguishing characteristics (e.g. low paid wages). These groups are more likely to be excluded from a number of social and community life aspects such as participation in civil society, lack of or inadequate provision of social goods (e.g. language services), education and healthcare, and exclusion from social production and consumption. As far as healthcare is concerned, immigrants usually face obstacles and difficulties in accessing the health services of the country of their residence as well as the quality of any services provided. European Union authorities have pinpointed the specific problem and have stressed the need for application of national policies for tackling it [7].

The health care system in Greece is linked to employment status and type of employment. The newly created National Health Services Organization was intended to cover the vast majority (more than 95%) of the population (workforce, dependents and pensioners), assuming the presence of only short-term unemployment. The basis for entitlement is insurance status. Regarding entitlement of migrants to health services, as stated in a study [8] conducted for the policies on health care for migrants in EU 27 “migrants legally residing in the country enjoy the same rights as citizens in terms of access to the healthcare system. The requirement however is to have insurance, as they cannot claim the welfare benefit, nor the card which allows persons with low income free access to healthcare. Free (or subsidized) healthcare is strictly connected to affiliation to a social insurance. Only legal aliens, namely those holding a residence and employment permit, have a right to social insurance”.

However, in the context of the deep crisis, unemployment rose rapidly since 2009 to reach 26.8% in January 2013. Under pre-existing legislation, EOPYY only effectively covers the unemployed for a maximum of two years, thus leading to a rise in the percentage of the uninsured population and migrants, as a vulnerable group, are mostly affected by that economic turmoil.

Even though scientists and health policies are continually dealing with the issue of inequalities in health care [9,10] there is not clear evidence about the health status of the approximately one million immigrants living in Greece today and their accessibility to healthcare services. Although immigrants are considered as a high risk group, vulnerable to poverty and social exclusion, there is a lack of an in-depth analysis and documentation of the factors that lead to this situation.

Until today, there hasn’t been a formed policy regarding the access and use of health care services mainly due to a lack of sound data for the epidemiological profile of immigrants and the use of health services by them. Therefore the development of any health care policy for immigrants must be evidence-based on health care needs of immigrants. Additionally, information is needed regarding the extent that the immigrants have access in health services and if that access is of satisfactory quality. Inevitably studies regarding the health literacy and use of health care services are essential.

This study aimed to explore the perceived barriers to access and utilization of healthcare services by immigrants in Greece. The present project aims to fill a gap by investigating the perceptions of immigrants from developing countries on barriers to healthcare access in Greece and proceed to specific proposals regarding the best ways to enhance the access of these groups in health care services.

## Methods

### Study population

A cross-sectional study was conducted from January to April 2012 in Athens, Greece. The study population consisted of 191 immigrants who were living in Greece less than 10 years. Although there is not a universally accepted definition of immigrant the term in the present study, is based on the definition as it is published in the Glossary of Migration [11] and it applies to persons, and family members, moving to another country or region to better their material or social conditions and improve the prospect for themselves or their family.

The participants reported being documented migrants. However, the investigators were not able to verify this information since immigrants often are reluctant or afraid to state differently. Regarding the sampling method, there is no accurate census of immigrants in Greece since, members of some immigrant populations, such as Pakistani and Indian for example, are extremely difficult to locate. Thus, probability or random sampling could not be realized and therefore snowball sampling (a non-probability sampling method) was applied. Country of origin of immigrants was Albania, Georgia, Afghanistan, Philippines, Russia, Bulgaria, Nigeria and Ghana. Initially, we contacted key persons in immigrant communities, such as their leaders or representatives. Those key persons acted as mediators between investigators and immigrants in order to increase feelings of trust and comfortability. Immigrants with a good level of Greek language proficiency facilitated the procedure of interviewing as translators. A workshop took place in order to inform the translators about the objectives of the study and to train them on the content of the questionnaire and the interviewing procedure. Face-to-face interviews were conducted with immigrants with a mean duration of 30 min in their native language. During the whole procedure both the investigators and the translators were present in order to ease the completion of the interviews. Through the abovementioned research design a high response rate (91%) was achieved. A little more than half of the participants (51.3%) were interviewed in their language, while 36.7% of the interviews were conducted in Greek and 12% in English. Participants were informed about the study and gave their written consent. Personal data of immigrants were not registered at any stage of the study. The study protocol was approved by the ethics committee of the Faculty of Nursing of the University of Athens.

### Measures

A questionnaire was developed including information on sociodemographic characteristics, health status, public health services knowledge and utilization and perception of difficulties in health services access. A qualitative study [12] was conducted prior to this one, in order to construct the quantitative questionnaire of our study. A pilot quantitative study with 30 immigrants was carried out, in order to improve the comprehensibility of the questionnaire. Internal consistency of the questionnaire was calculated by Cronbach’s alpha and was found equal to 0.7 which was considered acceptable.

Sociodemographic characteristics included age, country of origin, months of stay in Greece, gender, marital status, number of children, educational level (less than high school, high school, at least some college), smoking habits (smokers and non-smokers), health insurance coverage, employment at the time of study, family monthly income and living arrangement. Also, we measured immigrants’ ability to understand, speak, read and write in Greek in a five-point Likert-type scale (very poor, poor, moderate, good and very good).

Information about health status included self-reported health status, medication use for chronic diseases and existence of diagnosed hypertension, asthma, diabetes, cardiovascular disease, mental health disease, sexually transmitted disease and disease of digestive system.

Public health services knowledge was measured on a five-point Likert-type scale (very poor, poor, moderate, good and very good). For statistical analysis purposes, very poor and poor knowledge considered to be one category and also good and very good knowledge considered to be one category.

Public health services utilization included physician visits, dentist visits, use of emergency department services and inpatient hospital care. For physician and dentist visits, number of visits during the past 12 months was asked. For use of emergency department services and inpatient hospital care, the immigrants were asked whether they had been admitted in an emergency department or hospital within the preceding 12 months.

Difficulties in public health services access was measured on a five-point Likert-type scale (not at all, slightly, moderately, quite a bit and extremely difficult). For statistical analysis purposes, not at all and slightly difficult considered as one category and quite a bit and extremely difficult considered being one category also.

### Statistical analysis

The normality assumption was evaluated using Kolmogorov-Smirnov criterion (p>0.05 for all variables), histograms and normal probability plots. The continuous variables appeared reasonably normally distributed. Continuous variables are presented as mean (standard deviation, SD), while categorical variables are presented as absolute and relative frequencies. To determine associations between categorical variables, we used Pearson’s ×^2^ test and ×^2^ test for trend. Student’s t-test and Analysis of Variance (ANOVA) were applied for the analysis of group differences within continuous variables. Correlation between continuous variables was assessed with Pearson’s correlation coefficient. Due to multiple significance tests made (n=50), the Bonferroni correction was applied to account for the increase in type I error. So, a two sided p-value of less than 0.001 was considered statistically significant. The Statistical Package for Social Sciences (IBM SPSS) program, version 19.0 was used for statistical analysis.

## Results

The response rate was 91% (191 out of 210 questionnaires). Sociodemographic characteristics are presented in Table 1, information about health status is presented in Table 2 and their ability in Greek language in Table 3. Mean age of the study population was 37.4 years (10), while mean length of stay in Greece was 76.8 months (33.1). Mean number of children was 1.5 (1.2). More than half of the participants (56.5%) had health insurance coverage, 69.1% reported good/very good health status, while 14.7% reported medication use for a chronic disease. The ability of the participants to understand, speak, read and write Greek was 62.3%, 53.4%, 38.7% and 25.1% respectively.

**Table 1:** Sociodemographic characteristics of participants

**Characteristics**	**N (%)**
Gender	
Male	88 (46.1)
Female	103 (53.9)
Country of origin	
Albania	51 (26.7)
Georgia	42 (21.9)
Afghanistan	27 (14.2)
Nigeria, Ghana	25 (13.1)
Philippines	23 (12.0)
Bulgaria	12 (6.3)
Russia	11 (5.8)
Marital status	
Married	114 (59.7)
Single	69 (36.1)
Divorced/separated/widowed	8 (4.2)
Children	
Yes	134 (70.2)
No	57 (29.8)
Educational level	
Less than high school	60 (31.4)
High school	66 (34.6)
At least some college years	65 (34.0)
Smoking	
Yes	59 (30.9)
No	132 (69.1)
Health insurance coverage	
Yes	108 (56.5)
No	83 (43.5)
Employment at the time of study	
Yes	127 (66.5)
No	64 (33.5)
Family monthly income (€)	
0-250	35 (18.4)
251-500	16 (8.4)
501-750	42 (22.0)
751-1000	65 (34.0)
>1000	33 (17.3)
Pay for rent	
Yes	156 (81.7)
No	35 (20.3)

**Table 2:** Information about health status of participants

**Characteristics**	**N (%)**
Self-reported health status	
Very poor	0 (0.0)
Poor	4 (2.1)
Moderate	55 (28.8)
Good	98 (51.3)
Very good	34 (17.8)
Medication use for chronic diseases	
Yes	28 (14.7)
No	163 (85.3)
Hypertension	
Yes	8 (4.2)
No	183 (95.8)
Asthma	
Yes	10 (5.2)
No	181 (94.8)
Diabetes	
Yes	7 (3.7)
No	184 (96.3)
Cardiovascular disease	
Yes	4 (2.1)
No	187 (97.9)
Disease of the digestive system	
Yes	6 (3.1)
No	185 (96.9)
Mental health disease	
Yes	3 (1.6)
No	188 (98.4)
Sexually transmitted disease	
Yes	4 (2.1)
No	187 (97.9)

**Table 3:** Ability of participants in Greek language

**Degree of ability**	**Ability to**			
	**Understand Greek**	**Speak Greek**	**Read Greek**	**Write in Greek**
Very poor	3 (1.6)	5 (2.6)	18 (9.4)	27 (14.1)
Poor	8 (4.2)	12 (6.3)	39 (20.4)	52 (27.2)
Moderate	61 (31.9)	72 (37.7)	60 (31.4)	64 (33.5)
Good	76 (39.8)	60 (31.4)	47 (24.6)	27 (14.1)
Very good	43 (22.5)	42 (22.0)	27 (14.1)	21 (11.0)

Values are expressed as n (%).

Only 20.4% (n=39) of the participants reported that they had a good or very good degree of knowledge about public health services in Greece, most of them (n=115, 60.2%) reported that their knowledge was moderate and 19.4% (n=37) reported it as poor/very poor.

Increased ability to speak Greek was associated with increased health services knowledge (×^2^ test for trend=24.3, p<0.001) (Table 4).

**Table 4:** Statistically significant relations between sociodemographic characteristics and public health services knowledge

**Characteristics**	**Public health services knowledge**			**P value**
	**Poor/very poor**	**Moderate**	**Good/very good**	
Ability to speak Greek				<0.001^a^
Poor/very poor	12 (70.6)	4 (23.5)	1 (5.9)	
Moderate	11 (15.3)	55 (76.4)	6 (8.3)	
Good/very good	14 (13.7)	56 (54.9)	32 (31.4)	

Values are expressed as n (%).

^a^×^2^ test for trend.

A considerable proportion of the participants (n=119, 62.3%), needed at least once to use health services but they could not afford it, during the last year. The most important reasons for that were high cost of health care (n=41, 34.5%), long waiting times in hospital (n=15, 12.6%) and lack of free time (n=11, 9.2%).

Almost half of the participants (n=95, 49.7%) used public health services in the last 12 months in Greece. Among them, 56.8% (n=54) used emergency department services, 34.7% (n=33) visited physicians and 34.7% (n=33) visited dentists. Mean number of visits in emergency department services, physicians and dentists was 0.4 (0.7), 0.4 (1.2) and 0.4 (1.2) respectively. Twenty-five (13.1%) immigrants were hospitalized in the last 12 months in Greece with an average length of stay of 7.1 days (10.4).

There was not any statistically significant relationship between sociodemographic characteristics and use of public health services. More than half of the participants (n=101, 52.9%) reported that they had great difficulties in accessing health services, 29.8% (n=57) that they had moderate difficulties and 17.3% (n=33) that they had little or no difficulties. Increased family monthly income was associated with decreased difficulties in access to health services (×^2^ test for trend=32.1, p<0.001) (Table 5).

**Table 5:** Statistically significant relations between sociodemographic characteristics and access in public health services

**Characteristic**	**Difficulties in access in public health services**			**P value**
	**Quite a bit/extreme**	**Moderate**	**Not at all**/**slight**	
Family monthly income (euro)				<0.001^a^
0-250	28 (80.0)	4 (11.4)	2 (8.6)	
251-500	14 (87.5)	2 (12.5)	0 (0.0)	
501-750	27 (64.3)	11 (26.2)	4 (9.5)	
751-1000	28 (43.1)	26 (40.0)	11 (16.9)	
>1000	4 (12.1)	14 (42.4)	15 (45.5)	

Values are expressed as n (%).

^a^×^2^ test for trend.

Among the most important problems concerning public health services in Greece were long waiting times in hospitals (n=115, 60.2%), difficulties in communication with health professionals (n=87, 45.5%), high cost of health care (n=74, 38.7%) and system’s complexity (n=65, 34%).

Regarding their opinion about the quality of health care services provided to them, only 20.4% (n=39) of the participants reported that health services in Greece were good/very good, most of them (n=107, 56%) reported that health services were moderate and 23.6% (n=45) reported that health services were poor/very poor.

## Discussion

The findings of our study provide important information on the public health services knowledge and utilization among immigrants in Greece. We found that 56.5% of participants had health insurance coverage, a proportion relatively small compared to the natives [13]. This may be explained by the fact that immigrants either are unemployed, informally employed or undocumented (but were reluctant to state so) and therefore not able to apply for health insurance. Other studies in different immigrant populations also showed that they themselves as well as their children had less frequently health insurance coverage in comparison to native populations [14-16]. Private insurance coverage is also rarer among immigrants than natives [17,18]. Sixty-nine percent of immigrants reported good/very good health status, a proportion considered as satisfactory and explained by their young age. Previous studies conducted in Madrid [19], New York [20] and Amsterdam [21] confirm this finding. One possible explanation may be the “Healthy Immigrant Effect” since many studies have confirmed that immigrants have superior health status as compared to the native population [22-24].

Immigrants report better self-reported health and functional health than their native counterparts a phenomenon also been observed in many developed countries including Canada, Australia, the US and UK [25-27].

However, only 20.4% of the participants reported adequate knowledge of public health services in Greece in a good/very good degree while 52.9% reported having great difficulties in accessing health services. Previous research suggests that limited knowledge about health services decreases their utilization by immigrants [10,28,29].

Over half of the participants in the study (62.3%) expressed unmet needs regarding health care services. The most important reasons according to the respondents were long waiting times in hospitals, difficulties in communication with health professionals, high cost of health care and system’s complexity, findings also confirmed by other studies [30-37]. Access to care, is an essential element in achieving quality of life and growth, a main objective in the Health 2020 strategic plan [38]. Apart from the present study, there is a growing body of research acknowledging that immigrants face barriers in accessing health care services. Inaccessibility to health care services for immigrants represents an important concern for all host countries as it prevents newcomers from fully participating in society [22,39]. The delay in receiving the appropriate health care services or the unmet needs for health care services, may lead to an increased demand for in-hospital or emergency services having negative effects for the health outcome of the population, especially for the middle and the low-income households and the vulnerable groups such as immigrants which are the most affected [39-41]. Additionally, that may lead to higher health care expenditure in the long run, raising serious concerns about the Greek health system sustainability especially during the economic crisis. Policy makers should focus in increasing the efficiency of resource allocation and the preservation of access to health services regardless of wealth, education, age or ethnic group is imperative need.

Language barriers and miscommunication may result in the provision of suboptimal care and have an impact to medicine/treatment adherence [42,43]. According to our findings only half of the study population (53.4%) reported good or very good ability to speak while less than 39% reported good ability to read Greek. Good ability to speak the language was strongly correlated with health services literacy. This finding is in agreement with many studies since limited proficiency of the language of the host country has been associated with increased waiting times and has been identified as a significant obstacle in communication with health professionals, leading in inappropriate use of health services [44-46]. Moreover, limited proficiency of the language spoken at the host country decreases use of mental health services and immigrants’ satisfaction by the health services [47-50].

Based on our findings, the empowerment and facilitation of the health care access and provision for immigrants in Greece is necessary. Depending on the needs of the migrant population, simple measures such as comprehensive information regarding the available health services and the legal frame regarding the access for both lawfully residents and undocumented immigrants, is an important step towards enabling better access to needed services. Description of certain patient journeys in the system may also be proven useful. Health care professionals should be better trained in order to understand the special needs of the immigrants, the importance of accepting cultural differences and the right of immigrants to qualitative services. Public health policy measures related to appropriate coverage and adaptation of existing best practices should be taken in order the system to be better able to respond to increasing numbers of immigrants and assist in their social integration.

There are several limitations in our study. The study population was not a random sample of immigrants in Greece, although an effort was to include the major groups in proportions similar to those reported by recent statistical data. Additionally, our information on the health status and medical conditions was based on self-report and did not include objective measures, while the cross-sectional design of our study could not capture temporal changes in the ability of immigrants to use and access of health services. Also, data about utilization of health services were based on self-assessments for the 12 months leading up to the survey and therefore this information may be subject to recall bias. Larger scale studies including rural areas in Greece should be conducted to better understand health services knowledge and utilization among immigrants in Greece.

## Conclusions

Despite the limitations, this study offers an insight into an extremely important issue which constitutes subject of extensive discussions in Europe with a view to formulate a common policy. Among the main findings of this study was that the knowledge and use of public health services of immigrants was limited. Given the outbreak of new epidemics (e.g. influenza virus, H1N1) and the resurgence of infectious diseases which used to be in decline or elimination in Greece (e.g. tuberculosis and malaria), the fact that little is known about the health of immigrants and their access to proper health services, is concerning. Documentation regarding the immigrants’ health status, the needs and access to and use of health services is a crucial area of research. The findings of the study may contribute to tackle the phenomenon and stimulate further research, since there may be a step further to control and tackle the phenomenon. Co-ordinated action of governmental and nongovernmental organizations and researchers, based on best practices may succeed to improve access of immigrants to health services.

## Competing interests

All authors declared that they have no competing interests.

## Authors’ contributions

GP: participated in the design of the study, performed the statistical analysis and drafted the manuscript. SP: participated in the design of the study and performed the statistical analysis. BT: participated in the design of the study TM: participated in the design of the study and critical review the manuscript. KI: drafted the manuscript SO: participated in the design of the study CG: critical review the manuscript KD: participated in the design of the study and drafted the manuscript. All authors read and approved the final manuscript.

## Pre-publication history

The pre-publication history for this paper can be accessed here:

http://www.biomedcentral.com/1472-6963/13/350/prepub
